# Gene-Environment Interactions in Neurodevelopmental Disorders

**DOI:** 10.1155/2017/9272804

**Published:** 2017-01-31

**Authors:** Susanna Pietropaolo, Wim E. Crusio, Joram Feldon

**Affiliations:** ^1^University of Bordeaux, INCIA, Pessac Cedex, France; ^2^CNRS, INCIA, UMR 5287, Pessac Cedex, France; ^3^Department of Biology, The Swiss Federal Institute of Technology (ETH), Zurich, Switzerland

Neurodevelopmental disorders (NDDs) include a huge variety of pathologies characterised by varying degrees of intellectual disability and behavioural dysfunction. Research during the last years has underlined the genetic nature of the aetiological factors involved in most NDDs, identifying in some cases (e.g., Fragile X and Rett syndromes) single gene mutations as the unique pathological cause, while determining in others multiple genetic risk factors (e.g., schizophrenia, autism spectrum disorders (ASDs)). The high interindividual variability in several key pathological aspects of NDDs, such as the severity of the behavioural symptoms and the age-related progression, has motivated researchers to direct their attention to environmental factors that may critically influence the expression of the genetic “determinants” of these pathologies. This research has led to several theoretical models describing the relationships between genetic and environmental insults in NDDs: these models have in turn emphasised the additive or synergistic interactions between genes and environment and increased the interest in better understanding the specific contributions of these interactions in the aetiopathology of NDDs.

Gene-environment interactions are obviously of relevance to most disorders of the nervous system but are especially important for developmental pathologies, because of the considerable plasticity of the developing brain and its critical responsiveness to environmental changes. Indeed, a large body of human and animal data has demonstrated that environmental stimulation/deprivation can, respectively, ameliorate or exacerbate the symptoms of many NDDs. Nonetheless, animal studies combining both genetic and environmental manipulations are still scarce, at least compared to the huge amount of research work that concentrates only on genetic effects. This special issue aims to attract attention to the importance of gene-environment interactions, which so far have often been ignored. Review and original research articles are combined to discuss the impact of the interactions between genetic and environmental interventions in both clinical and preclinical studies. A variety of NDDs are included, such as ASDs, schizophrenia, Fragile X, Down syndrome, and ADHD, and a multitude of genetic and environmental manipulations are discussed. Several environmental factors were studied, such as nutritional manipulations, immunological changes, environmental enrichment, stress, or social deprivation. These sometimes implied environmental adversity, but in other cases environmental stimulation, possibly supporting nonpharmacological therapies based on sensory-social or nutritional enrichment.

The review by C. Madore et al. investigates the relevance of nutritional factors in the aetiopathology of neurodevelopmental disorders. This article is focused in particular on the role of polyunsaturated fatty acids (PUFAs), because of the association between imbalances in PUFA levels and NDDs. Preclinical and clinical data are summarised, highlighting the anti-inflammatory properties of PUFAs and their impact on the microbiota, which is suggested as the main factor potentially linking inflammation, environmental adversity, and NDDs. P. Moran et al. instead provide a review of preclinical studies on gene-environment interactions in schizophrenia using genetic animal models. This review discusses the synergistic effects of genetic and environmental risk factors on the expression of those endophenotypes that are relevant to schizophrenia, thus supporting the validity of multifactorial preclinical models.

The research article by E. Aronoff et al. provides original clinical data on the therapeutic impact of environmental enrichment in autistic children. Indeed, a therapy based on daily sensory enrichment is shown to ameliorate children's behavioural problems in multiple domains, including cognitive, emotional, social, and sensorial abilities. Interestingly, these effects were largely independent of the subjects' gender, nationality, or initial severity levels of the pathology. The article by L. Garbugino et al. parallels this human study, providing original data on the effects of early enrichment in an animal model of social dysfunction, the KO mouse for the *μ*-opioid receptor gene (*Oprm1*^−*/*−^). Early environmental stimulation of the highly immature mouse pups using an enrichment protocol providing additional maternal care was applied and both short- and long-term behavioural effects of this manipulation were detected. The research article by E. Burrows et al. focuses its attention instead on environmental adversity, evaluating the behavioural effects of social isolation housing in a mouse model for autism, the Neuroligin-3 (NL3) mouse. The data presented here also highlight the importance of the choice of the procedures for behavioural testing in research on genetic models of neurodevelopmental disorders, since the effects of the environmental and genetic manipulations differed depending on the specific social test used.

The review by I. De Toma et al. expands the evaluation of the effects of gene-environment interactions in NDDs by assessing the role of epigenetic mechanisms in the aetiopathology of these diseases. The link between global and local epigenetic alterations, such as anomalies in DNA methylation, histone modifications or chromatin remodelling, and developmental disorders, is discussed, with a focus on two examples of developmental pathologies characterised by cognitive impairments, Down syndrome (DS) and Fragile X syndrome (FXS). This article also directly describes therapeutic approaches using epigenetic drugs that can act as cognitive enhancers in DS and FXS.

In conclusion, this special issue emphasises the importance of continuing and extending neurobehavioural studies on NDDs combining genetic with environmental manipulations and suggests that a multifactorial approach is most likely to identify novel therapeutic approaches and to advance our understanding of the aetiopathology of these complex disorders.

*Susanna Pietropaolo* *Wim E. Crusio* *Joram Feldon*

